# Assuring health insurance policies for better health outcomes and financial stability of transgender community during the monkeypox disease outbreak

**DOI:** 10.1097/JS9.0000000000000027

**Published:** 2023-03-24

**Authors:** Vishnu Priya Veeraraghavan, Jyotsna Needamangalam Balaji, Sreenidhi Prakash, Ullas Mony, Krishna Mohan Surapaneni

**Affiliations:** a Centre of Molecular Medicine and Diagnostics (COMManD), Saveetha Dental College and Hospitals, Saveetha Institute of Medical and Technical Sciences, Saveetha University; b Panimalar Medical College Hospital & Research Institute; c SMAART Population Health Informatics Intervention Center, Foundation of Healthcare Technologies Society, Panimalar Medical College Hospital & Research Institute; Departments of d Biochemistry; e Medical Education; f Molecular Virology; g Research; h Clinical Skills & Simulation, Panimalar Medical College Hospital & Research Institute, Chennai, Tamil Nadu, India

*Dear Editor*,

The steps towards inclusivity of transgender community in prevention of monkeypox disease outbreaks and health management are extremely important and critical as well. Understanding of the paucity in gender identity data that makes the prediction as well as estimation of monkeypox disease outbreak and the transmissibility is very poor among the transgender and gender minority people^1^.

Along with providing the global scenario of monkeypox disease prevalence, predisposition among transgender women, and the mitigation steps that has to be taken to prevent the transmission of monkeypox, the article also stresses the consideration of the financial needs of these vulnerable groups. Thus, recognising its importance, we take this opportunity to emphasize on the need to devise appropriate health insurance policies that will assure economical support to the socially discriminated communities during the times of crisis.

Globally, the rate of gender minority people being health insured is relatively lower than the general population^2^. In India, owing to the verdict of Supreme Court in 2014, the Life Insurance Corporation has included the “3rd gender option” in its health insurance forms. Yet, the nationwide inclusivity of transgender community in all insurance companies is still suboptimal^3^. Hence, as stated by the authors the inconsistency and nonstandardization in collection, storage and retrieval of gender identity data makes the estimation of insured and noninsured transgender people challenging leaving behind a large group of the population unnoticed. The recent ‘COVID-19 pandemic’ has caused much of physical, mental, and financial burdens on people all over the world. Among them, the gender minority population has suffered drastically due to inequalities and inaccessibility to healthcare facilities and health-related policies that have resulted in severe health and economic instabilities^4^. Discrimination, lack of government-assured health policies, restriction in job recruitments caused financial crisis during isolation and has resulted in devastating health conditions, exploitation, and high suicidal rates among transgender people^5^. Thus, the need for proper health insurance policies to provide economic support to the vulnerable people becomes the need of the hour.

Owing to the recent surge in monkeypox infection rates, precautionary measures are being taken worldwide to prevent another catastrophe. This also includes supporting and safeguarding the underprivileged gender minority groups through health insurance plans and disease management protocols as they are at a higher risk of acquiring the infection. Unfortunately, sex workers, HIV-infected patients, transgender, and other gender minority population are still being given least importance in terms of nonprioritisation and lack of access to vaccination and health insurance schemes. This stresses the immediate need to avail this population with the health rights that they deserve to reduce the harmful impacts of another possible outbreak and to eliminate the trend of gender minority people being the first to get affected and last to be protected^6^. Therefore, In addition to inclusion in preventive and therapeutic strategies, we also insist the health insurance inclusion and full-fledged adoption of the scheme for the transgender community. This will undoubtedly transform the lives of these people by promoting adequate health and financial benefits thereby improving their lifestyle in a nondiscriminative and welcoming environment.

## Ethical approval

Not applicable.

## Sources of funding

None.

## Authors’ contribution

V.P.V.: conceptualization, data curation, writing – original draft preparation. J.N.B., S.P., U.M.,: data curation, writing – original draft preparation. K.M.S.: conceptualization, study design, data curation, writing – original draft preparation, reviewing and editing, visualization and supervision.

## Conflicts of interest disclosure

The authors declare that they have no financial conflict of interest with regard to the content of this report.

## Research registration unique identifying number (UIN)

None

## Guarantor

Krishna Mohan Surapaneni.
